# Engineering Dual p–n-Type CuI with Significant Enhanced Performance for Advanced Thermoelectric Applications

**DOI:** 10.1021/acsaem.4c03130

**Published:** 2025-01-27

**Authors:** Mustafa Majid Rashak Al-Fartoos, Anurag Roy, Tapas Kumar Mallick, Asif Ali Tahir

**Affiliations:** Solar Energy Research Group, Department of Engineering, Environment and Sustainability Institute, University of Exeter, Penryn Campus, Penryn, Cornwall TR10 9FE, United Kingdom

**Keywords:** CuI, doping, glazing, p−n type, nanogenerator, renewable, semiconductor, thermoelectric, window

## Abstract

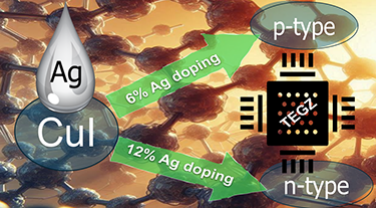

CuI is a well-known thermoelectric (TE) material recognized for its p-type characteristics. However, the development of its n-type counterpart and the integration of both p- and n-type CuI in thermoelectric generators (TEGs) remain largely unexplored. In this study, we successfully tuned the thermoelectric properties of CuI by strategically incorporating Ag, enabling the synthesis of both p-type (Ag_0.2_Cu_0.8_I) and n-type (Ag_0.9_Cu_0.1_I) materials using a cost-effective, greener, and scalable successive ionic layer adsorption and reaction (SILAR) method. The p-type Ag_0.2_Cu_0.8_I exhibited a figure of merit (ZT) of 0.47 at 340 K, driven by a high Seebeck coefficient of 810 μV·K^–1^. In contrast, the n-type Ag_0.9_Cu_0.1_I achieved an exceptional ZT of 2.5 at 340 K, attributed to an ultrahigh Seebeck coefficient of −1891 μV·K^–1^. These superior thermoelectric properties make CuI-based materials attractive alternatives to conventional TE materials, such as Bi_2_Te_3_ and PbTe, which are limited by toxicity and resource scarcity. Furthermore, a prototype thermoelectric glazing unit (5 × 5 cm^2^) demonstrated a 14 K temperature differential, highlighting its dual functionality in power generation and building heat loss mitigation. These findings underscore the potential of low-cost CuI-based materials for advancing sustainable energy technologies.

## 1 Introduction

Thermoelectric (TE) materials are vital for sustainable energy, converting waste heat into electricity, and aiding climate change mitigation. They provide a dual benefit by generating renewable energy and enhancing energy efficiency through the Seebeck effect.^1^ Because there are no moving components, TE devices are simple to use, compact, and quiet and require no maintenance. However, TE materials generally suffer from low efficiency, and traditional high-efficiency materials like Bi_2_Te_3_ and PbTe are often expensive, toxic, or derived from rare resources. Despite this, ongoing interest in TE devices has driven the development of various TE materials and efforts to enhance their performance. However, the integration of these materials into glazing systems, particularly window glazing units, remains an underexplored concept. This study explores the potential of incorporating thermoelectric glazing (TEGZ), which can both harvest waste heat and improve energy efficiency. TEGZ offers dual functionality, enabling power generation while enhancing thermal regulation, thus contributing to energy generation and improved thermal management in buildings.^2,3^

Consequently, windows are a crucial part of buildings, because they provide daylight, indoor comfort, solar heating gain, and ventilation. They are the connections between the interior space of the building and the exterior. However, windows are often regarded as the weakest part in a building’s energy efficiency, accounting for 40% of global energy consumption and contributing to 40% of total energy losses in modern structures.^4,5^ Improving window energy efficiency through TEGZ could significantly reduce these energy losses.

There are many attempts to increase the energy efficiency of windows by reducing heat losses via using double glazing, triple glazing, low-emissivity coating, water flow glazing, airflow glazing, vacuum glazing, aerogel glazing, and prismatic glazing. Moreover, there are attempts to increase windows’ energy efficiency by converting the glasses into a power source by implanting photovoltaics (PVs) into the windows.^6,7^ However, PV could increase heat losses and require light to generate power. TE can be integrated with glazing to reduce heat losses and generate power without needing light.^8^ TE materials usually have low thermal conductivity (*k*), which makes them suitable as thermal insulators that could reduce heat losses. Also, it can work with or without daylight and its performance cannot be affected by dust and soiling. Additionally, TE materials offer the benefit of transparency and the feasibility of large-scale production. Finally, TE materials can work simultaneously with PV and can be combined with another type of thermal insulation glazing system.

To meet the requirements of TEGZ, TE materials should not only perform well at room temperature but also be transparent, cost-effective, and made from nontoxic, earth-abundant materials. TE materials that are used in TEGZ should have high performance at room temperature to meet the glazing application working temperature. The efficiency of TE materials is quantified using a dimensionless metric known as the figure of merit (*ZT*). *ZT* is directly proportional to the squared Seebeck coefficient (*S*) and electrical conductivity (σ) while being inversely proportional to *k*. *S* and σ together create the power factor (PF = *S*^2^σ).^9^ For example, Novak et al. achieved a high PF of over 600 μW m^–1^ K^–2^ at room temperature for both n-type and p-type graphene TE films by changing the adsorbed surfactant during the intercalation–exfoliation process.^10^ Moreover, TE materials should be transparent to satisfy glazing application. For example, Wang et al. fabricated a thermoelectric generator (TEG) with high transparency of around 81% in the visible-wavelength range.^11^ TE materials should be cost-effective and made from nontoxic earth-abundant materials. For example, Yang at el. fabricated transparent TE materials made from abundant nontoxic CuI and achieved 0.23 *ZT* at 300 K.^12^ TEGZ should be fabricated on a large scale to fulfill the requirements of a large area of windows. For example, a process of chemical batch has been used to convert single-crystalline Bi_2_Te_3_ nanowires into jettable ink to make them able to print on a glass substrate.^13^ Klochko et al. prepared semitransparent p-CuI and n-ZnO thin films by the successive ionic layer adsorption and reaction (SILAR) synthesis method. SILAR is considered as a cost-effective method for depositing materials layer by layer with precise control over thickness and composition at a low temperature.^23^

There have been some efforts to fabricate TEGZ.^8^ For example, a TEGZ with a 132.25 cm^2^ area was fabricated from Plexiglas panels. The TEGZ had a 6 mm thickness with 72 pairs of holes. Each pair was inserted with a p-type (Bi_0.4_Sb_1.6_Te_3_) and n-type (Bi_1.75_Te_3.25_) thermopile. The TEGZ produced an output power of 0.16 μW at a 22.5 K temperature difference.^14^ However, the TEGZ was made from expensive, rare materials and was fabricated by a complicated process. Moreover, a TEGZ with an area of 100 cm^2^ was fabricated by coupled 12 Bi_2_Te_3_-based TEG around glass that was coated by wavelength-selective coating. This coated glass was 88% transparent and able to absorb heat from ultraviolet and infrared light. When the TEGZ was exposed to sunlight, the temperature of the glass increased, which in turn raised the temperature of the mounted TE devices, resulting in a voltage output of 4 V.^15^ However, TEGZ has many drawbacks; for instance, it absorbs heat, which leads to an increase in the temperature of the buildings, and it is made from expensive and rare materials. Furthermore, a TEGZ made from ZnO was fabricated by a simple large-scale synthesis method (electrochemical deposition).^16^ However, this TEGZ had a low efficiency. Additionally, a TEGZ made by RF magnetron sputtering from CuI n-type and GZO p-type had a transparency of 70% gain in output power of 0.46 nW at a 13 K temperature difference.^17^ However, the TEGZ fabrication method was very complicated, and the TE material synthesis was performed by an expensive method. Many TEGZ designs rely on costly, rare materials and complex fabrication, while alternatives like CuI face low efficiency. Therefore, it is crucial to use these abundant materials and focus on improving their efficiency.

Considerably, earth-abundant, less toxic materials CuI and AgI are well-established materials in TE research, with their properties extensively studied in various forms, including doping, composites, and individual compounds. However, alloying CuI and AgI into a single material presents an innovative approach to enhancing the TE performance. This strategy aims to fine-tune the TE properties through the synergistic combination of CuI and AgI, offering potential for improved efficiency. Such an approach not only introduces a novel way to optimize performance but also reduces material consumption and enables a streamlined integrated synthesis process, making this a compelling area of this study. In the Ag–CuI thin film system, the similarities between Cu and Ag are leveraged as they belong to the same group. The use of ternary alloy formation strategies for semiconductor materials allows for the electricity manipulation and transmittance, including tuning of the optical band gap. Examples like InGaAs, MgZnO, and CdSeTe systems have demonstrated successful property engineering through ternary alloy formation with applications in optoelectronics. By using ternary alloys as semiconductors, it is possible to enhance the electricity and transmittances and tune the optical band gap. On the other hand, Ag–CuI is regarded as a transparent conductive material, offering a notable advantage over CuI due to its improved σ.^18^ This enhancement in electrical properties makes Ag–CuI a promising candidate for advanced applications where both transparency and high conductivity are essential.^19^ The charge carrier conductivity type of this material can be altered from n-type to p-type through adjustments in the Ag/Cu ratio.^20^ Synthesis methods include magnetron sputtering,^20^ mechanochemical techniques,^21^ and melt-annealing techniques; however, they are intricate and costly. Both p-type and n-type semiconductor materials have been synthesized by synthesis radio frequency sputtering from CuI by varying the Ag doping concentration.^20^ The effect of Ag doping on the TE properties of CuI has been conducted, and it was found that the *S* of Ag–CuI alloy increased up to 90% compared to undoped CuI. Moreover, the *k* reduced with the increase of Ag doping due to phonon scattering.^22^

**Figure 1:**
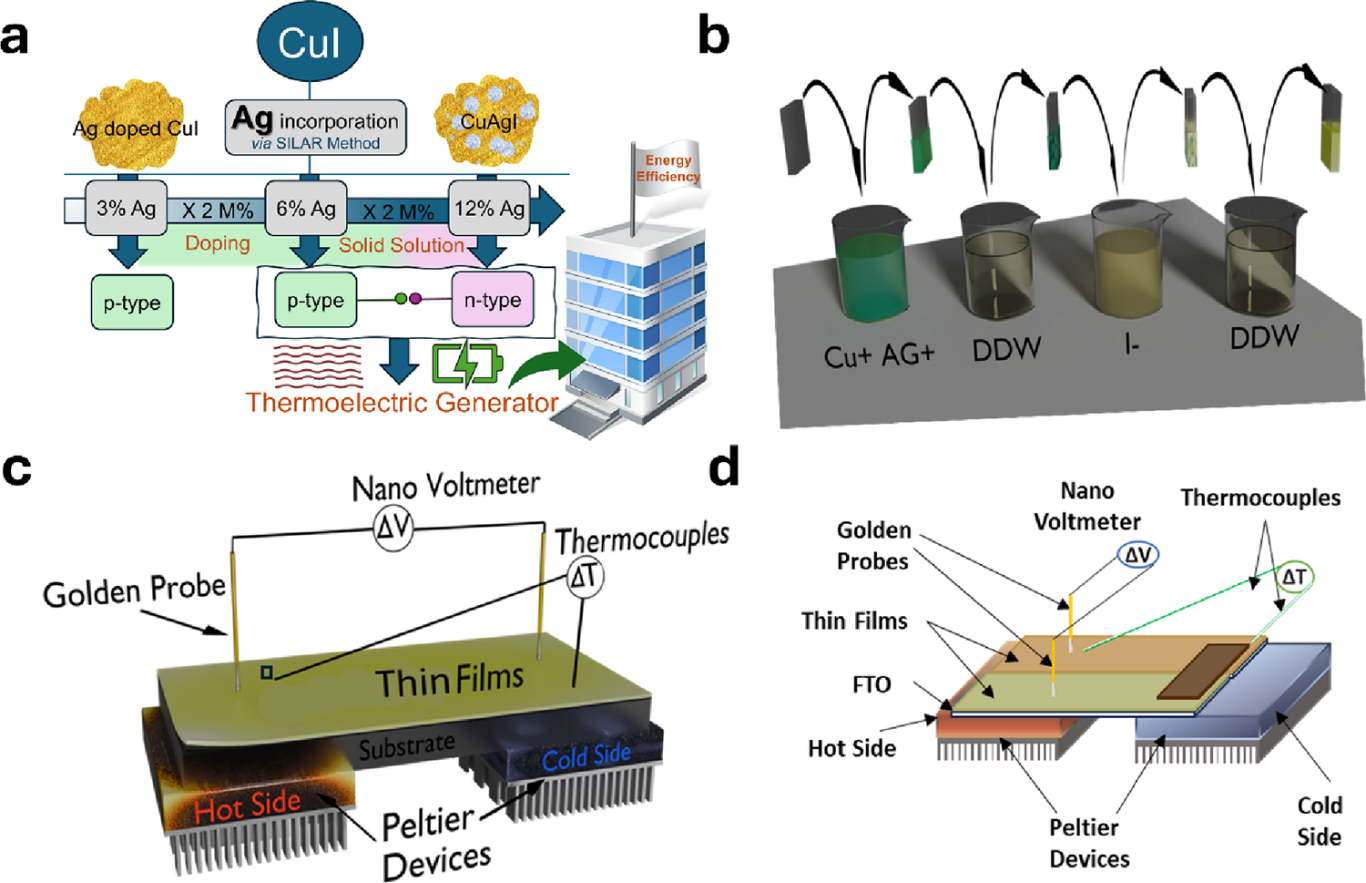
(a) Schematic illustration of the overall workflow and objectives of this research. (b) Diagram of a single SILAR cycle used for material synthesis. Schematic representation of (c) a custom device design for measuring the in-plane Seebeck coefficient and (d) the corresponding power output measurement setup for the thermoelectric nanogenerator.

In this work, we adopted a comprehensive approach to develop a high-efficiency TEG by using a simple, cost-effective synthesis method at room temperature. The TEG employs Ag–CuI thin films as n- and p-type materials, deposited on conductive glass substrates via the efficient SILAR method. Notably, through controlled Ag doping and the straightforward SILAR technique, CuI exhibits both n- and p-type properties. This dual functionality offers a novel approach, enabling the use of a single-material system to develop TEGZ. The overall research framework of this study is illustrated in Figure 1a, which provides an overview of the methodology and key steps. This approach simplifies the fabrication process and reduces the time required, making it a promising strategy for advancing efficient and scalable TEGZ technologies. A thorough analysis was conducted to assess the TE properties, and the generator’s performance was systematically measured and analyzed. Additionally, a detailed examination of the temperature gradient across a 5 × 5 cm^2^ TEGZ was performed to gain insights into its thermal behavior. The results demonstrate the effectiveness of Ag-doped CuI materials in achieving synergistic n- and p-type conductivities, offering a pathway for efficient TEG fabrication. This innovative approach is particularly promising for applications such as TEGZ, contributing to advancements in energy-efficient building technologies and material development strategies.

## 2 Materials and Methods

### 2.1 Materials

AgCl, CuSO_4_, Na_2_S_2_O_3_, and NaI were purchased from Thermo Scientific, UK. Fluorine-doped tin oxide (FTO) conductive glasses (NSG TEC 15) with a sheet resistance of 12–14 Ω·cm^–2^ were sourced from Pilkington. All chemicals were used as received without further purification.

### 2.2 Synthesis of the CuI Thin Film

A CuI thin film was deposited onto FTO glass using the SILAR method, following a process like that described in refs (23,24). All used FTO substrates were cleaned before the deposition process via ultrasonication in distilled water, isopropanol, and acetone, followed by drying. Figure 1b illustrates the SILAR process that utilized four beakers. Beaker A contained an aqueous Cu(I) thiosulfate complex solution (Na[Cu(S_2_O_3_)]) prepared by mixing 0.1 M CuSO_4_, 0.1 M Na_2_S_2_O_3_, and 0–0.012 M AgCl, serving as the cationic precursor. Both beakers B and D held Milli-Q water for rinsing, and beaker C contained a 0.075 M aqueous NaI solution, serving as the anionic precursor. The deposition cycle involved dipping the substrate into beaker A for 20 s, allowing a monolayer of Cu^+^ and Ag^+^ ions to adsorb onto its surface. Next, the substrate was rinsed in beaker B for 10 s to remove excess ions. This was followed by immersion in beaker C for 20 s to enable a reaction between Cu ions and iodine. Finally, the substrate was dipped into beaker D for 10 s to eliminate loosely attached particles and ions. The cycle was repeated 40 times for each thin film. Once the deposition cycles were completed, the substrate was air-dried for at least 4 h and subsequently annealed at 423 K. The SILAR process was performed using an integrated, software-controlled Ossila Dip Coater.

### 2.3 Material Characterization

The structural, morphological, and compositional properties of SILAR-synthesized Ag–CuI thin films were characterized by using advanced analytical techniques. The crystal structure was examined with a Bruker D8 Advanced XRD system, which provided insights into the phase purity and crystalline nature of the films. Nanostructural morphology was investigated by using a focused ion beam-scanning electron microscope (FIB-SEM, XT Nova NanoLab 600). The chemical composition was analyzed with energy-dispersive spectroscopy (EDS) by employing an Oxford Instruments X-MaxN system.

Film thickness measurements were conducted using a Bruker Innova atomic force microscope (AFM), ensuring precise thickness profiling. High-resolution transmission electron microscopy (HRTEM) and selected area electron diffraction (SAED) were performed with a JEOL JEM-2100F TEM operating at 200 kV, providing detailed imaging of the microstructure and crystallographic features. Additionally, an atomic absorption spectrometer (AAS) (Agilent 240 FS) was utilized to accurately quantify the elemental composition.

The optical properties, including the energy band gap, were determined using absorbance spectra analyzed via Tauc’s equation, as detailed in prior studies.^25,26^ X-ray photoelectron spectroscopy (XPS) analysis was conducted on a Thermo Nexsa XPS equipped with a monochromated Al Kα X-ray source (1486.7 eV) and a dual-beam charge neutralizer. Survey scans were performed at 200 eV pass energy, and high-resolution scans at 40 eV, under <10^–8^ Torr at 294 K. The ion gun was operated at 150 μA and 45 V. Calibration was based on the C 1s peak at 284.8 eV, and data were processed using CasaXPS v2.3.20PR1.0.

Four-point probing was used to measure σ of the thin film. The in-plane *S* of the thin films was measured by dividing TE output voltages onto temperature gradients (Δ*T*) along the film, as previously described in refs (16,17,27,28). The thin film was placed on two Peltier devices, one used as a hot source and one as a cold source. The output voltage was measured through two golden probes placed on the film, one placed on the hot side and another one on the cold side, while temperature differences were measured by two thermocouples placed on hot and cold sides as shown in Figure 1c. Prior to each measurement, the *S* measurement system was calibrated using pure aluminum and copper plates, following the methodology described in a previous study.^28^

The *k* measurements were performed using a C-Therm Trident system with accuracy of five readings on average. Moreover, the power output of the TEGZ that consists of p-type and n-type deposits on FTO was measured by placing it on two Peltier devices used as a hot and cold source. A nanovoltmeter was used measured the output voltage and current through the two probes placed on the p-type and n-type of the thin films. Two thermocouples were employed, one placed on the surface of the hot side of TEGZ and another on the surface of the cold side, to measure the temperature differences, as shown in Figure 1d.

### 2.4 Formulas Used for TE Performance Evaluation

The total *k* of a material is composed of two main components: the lattice thermal conductivity *k*_l_ and the electronic thermal conductivity *k*_e_, as shown in eq 1.^29^
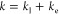
1

The electronic contribution can be calculated using the formula, as shown in eq 2:
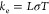
2where *L* is the Lorenz number, typically 1.5 × 10^–8^ for nondegenerate semiconductors.^30^

The power output of the TEGZ, current–voltage (*I*–*V*), and current–power (*I*–*P*) characteristic curves were calculated at different temperature gradients in the n–p module TEGZ. The open-circuit voltage (*V*_OC_) was calculated according to eq 3 described by^12^
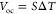
3the voltage output (*V*_out_), which is the voltage at the load resistance terminals to obtain the maximum power (*P*_out_), was calculated using eq 4:^17^
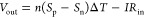
4where *n* is the number of TE elements, *S*_p_ is the *S* of the p-type, *S*_n_ is the *S* of the n-type, and *R*_in_ is the internal resistance of the TE element.

Finally, the output power is calculated by eq 5:
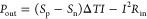
5

## 3 Results and Discussion

### 3.1 Microstructural Characterizations of the Ag–Cu–I Thin Film

Three different phases are distinct in CuI: α-CuI, β-CuI, and γ-CuI. Notably, the γ phase is the most stable phase at ambient temperature.^31^ Utilizing XRD, it was determined that the γ-Cul thin film deposited on FTO substrates possesses a crystalline structure. This pattern is closely similar to the established γ-Cul profile shown in the literature (JCPDS card no. 82-2111).^32^ Remarkably, XRD analysis exhibits distinct peaks indicative of a well-defined crystalline structure, albeit with slight broadening attributed to the nanoscale dimensions of the synthesized material. Notably, across variations in experimental parameters, all XRD diffraction peaks consistently correspond to γ-CuI, with no evidence of impurity phases.

**Figure 2:**
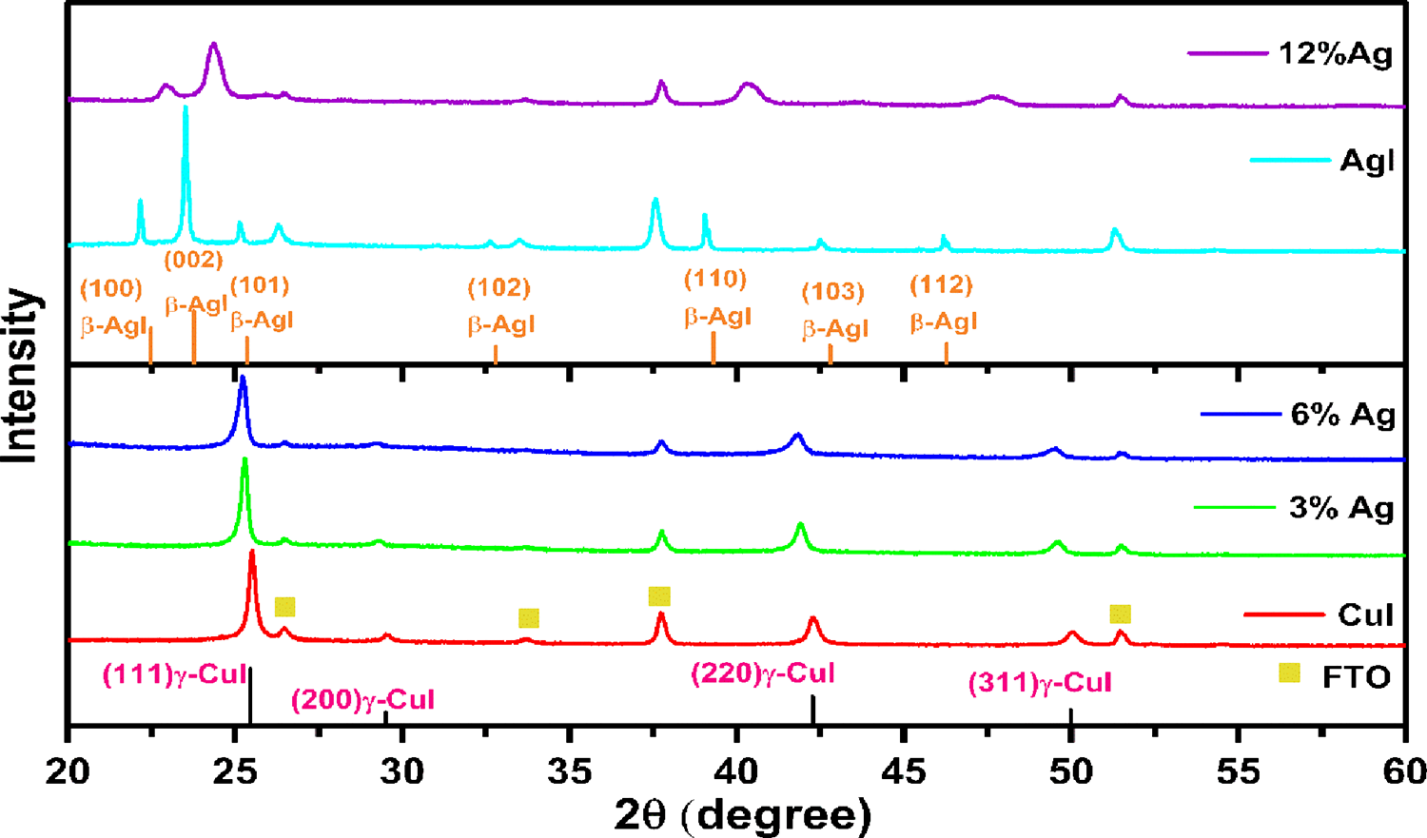
XRD patterns comparing various Ag−CuI thin films with their corresponding native samples.

The XRD data analysis of CuI showed a face-centered cubic (FCC, space group *F*4̅3*m*) structure with an average lattice parameter of 6.04 Å. The specific 2θ values (25.51, 29.52, 42.29, and 50.08°) are shown in Figure 2. Notably, the XRD pattern revealed that the (111) diffraction peaks exhibited the highest intensities. Applying the Scherrer equation,^33^ it was observed that the diameter of CuI crystallite sizes (coherent scattering domains) reduced gradually from 26.9 to 14.32 nm with the increase of Ag addition, as shown in Table 1, which led to a reduction in the intensities, indicating the successful doping of Ag within the CuI lattice.^33^ Doping with Ag caused a shift of peaks to lower angles, indicating the substitution of Cu atoms by Ag in the cubic γ-CuI phase, with no change in the XRD pattern as Ag doping increased from 3 to 6%.

**Table 1:** Comparative Analysis of the Crystallite Size and Lattice Parameters for Ag–CuI Thin Film Samples

thin film	unit cell	crystallite size (nm)	lattice parameter (Å)
CuI	cubic	26.97	*a* = 6.04
Ag (3%)	cubic	26.45	*a* = 6.09
Ag (6%)	cubic	25.25	*a* = 6.11
Ag (12%)	hexagonal	14.32	*a* = 4.02, *c* = 7.29

Incorporation of 12% Ag into the CuI lattice results in a single phase and has unique supersaturated structures, rather than a composite or simple doping, as evidenced by XRD analysis. This incorporation induces significant lattice elongation, triggering a structural transformation from the cubic γ-CuI phase to the predominantly hexagonal (wurtzite) β-AgI phase. The transition from cubic to hexagonal symmetry is characterized by the progressive evolution of γ-CuI → β-AgI phases. Notably, this transformation proceeds as a single-phase transition, without the emergence of secondary phases, signifying the development of a pseudobinary solid solution within the Ag–CuI system.^34^

The complete miscibility of Cu and Ag in the solid solution is further corroborated by the absence of distinct XRD peaks associated with separate AgI or CuI phases. Instead, the observed peak merging and broadening reflect a uniform pseudobinary structure, indicative of the successful integration of Ag and Cu within a single-phase matrix. Additionally, this solid solution formation is accompanied by a 43% reduction in crystallite size as the Ag content increases to double. The smaller ionic radius of Cu^+^ (145 pm) relative to Ag^+^ (165 pm) contributes to this size decrement. The detected diffraction peaks of the synthesized particles exhibit significant broadening and weakening, attributable to the presence of nanocrystals and supersaturated Cu atoms.^35^ Consequently, the peak shifts caused by lattice distortion are obscured by the broadened and diminished diffraction signals as the Cu content increases. This phenomenon underscores the effective incorporation of Ag into the CuI lattice, resulting in a homogeneous, single-phase pseudobinary solid solution system. Additionally, the structure transformed to the β-AgI crystalline phase, where no more CuI is expected to be observed. The XRD pattern of β-AgI matches with the standard reported in JCPDS card no. 046-1205.^34^

**Figure 3:**
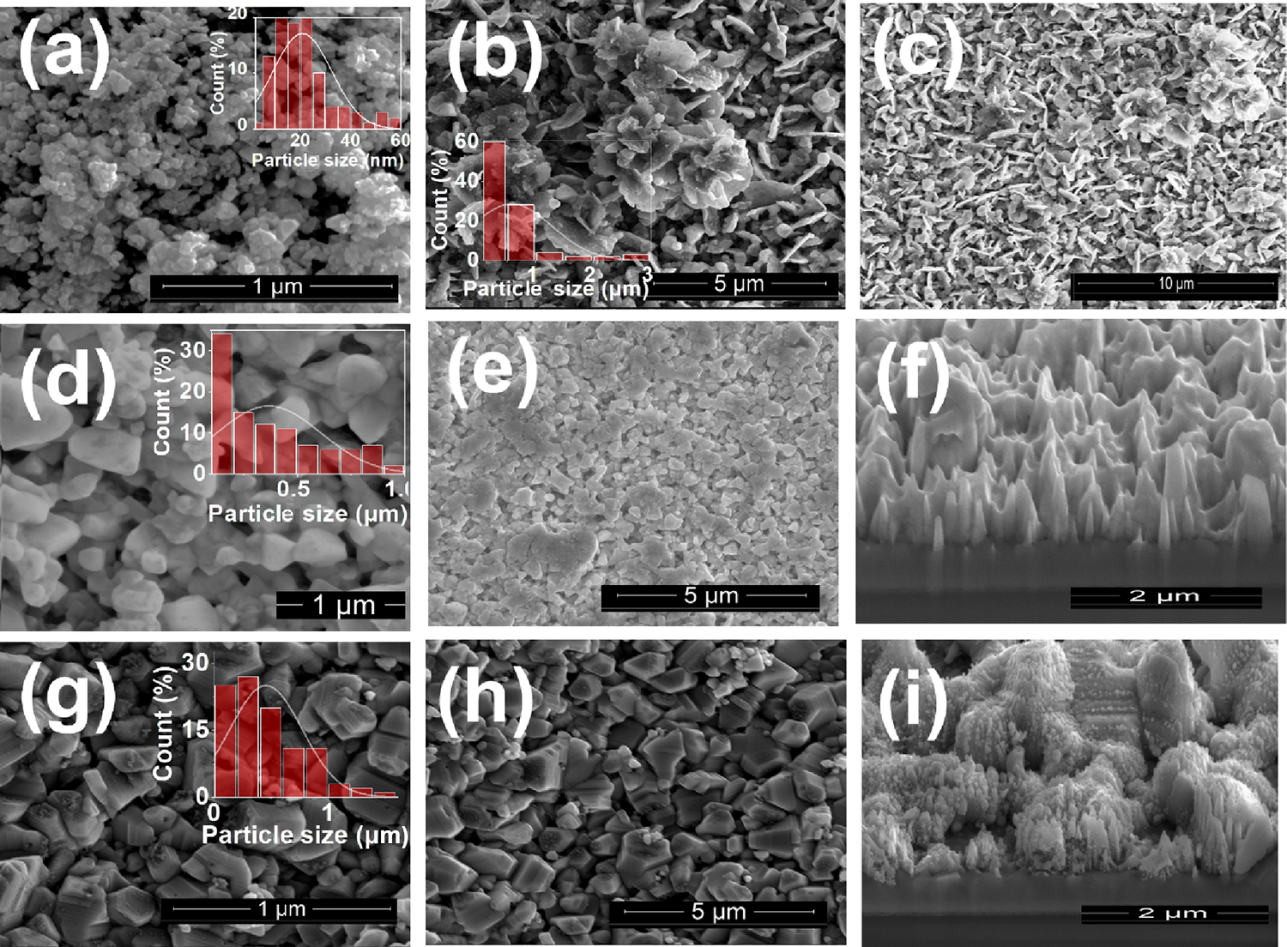
SEM micrographs showing the effect of varying Ag concentrations on Ag–CuI thin films. (a) Pure CuI. (b, c) Ag (3%)-CuI at different magnifications. (d, e) Ag (6%)-CuI thin films at different magnifications. (f) FIB-SEM image illustrating the film thickness of Ag (6%)-CuI. (g–i) SEM microstructures of Ag (12%)-CuI, at different magnifications.

Scanning electron microscopy (SEM) images in Figure 3a–i demonstrate the morphology of the synthesized Ag–CuI films, revealing a rough CuI surface with deformed particles that form like clustered grapes with cubic particle structures that are relatively much smaller in size (<40 nm), as shown in Figure 3a. In Figure 3b,c, when the Ag doping is 3%, the particle immerses and its shape is converted into plates with some parts of these plates merging into flower-like shapes. The surface stays highly rough and irregular, exhibiting an inhomogeneous texture. However, this roughness progressively decreases with increasing Ag doping. Notably, a doping level of 6% Ag (Figure 3d,e) results in a significant increase in particle size, from 20 nm in pure CuI to approximately 360 nm with 6% Ag. This indicates that higher Ag concentrations promote particle growth within the film, with the average particle size reaching 450 nm. This is due to many reasons: for example, increasing Ag content will lead to Ostwald ripening, where smaller particles merge into larger ones for thermodynamic stability.^36^ Ag incorporation also reduces nucleation sites, allowing existing particles to grow larger, and the lattice strain due to differences in ionic radius between Cu and Ag atoms promotes strain relaxation, further encouraging particle growth. Moreover, Figure 3f exhibits a 3d FIB-SEM image of Ag 6% with longitudinal growth. Furthermore, when the Ag addition increases to 12%, it results in a growth of cluster-type disordered hierarchical structures with an average diameter of 300 nm in the direction of [001], as shown in Figure 3g–i.^37^ The orientation of cluster-type disordered hierarchical structures may contribute to the observed low *k*. The stacked clusters inherent to these structures enhances phonon scattering and minimizes the contact surface area, thereby impeding effective heat transfer.^27^

**Figure 4:**
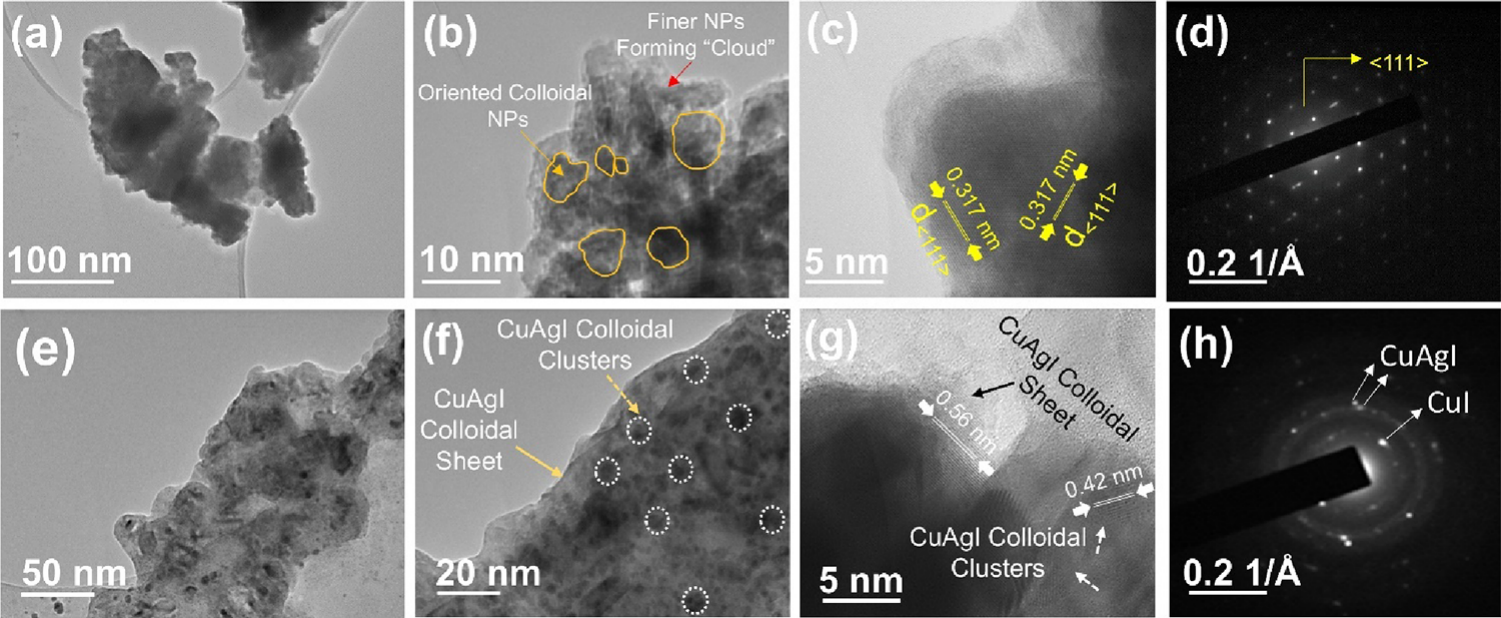
Transmission electron microscopy (TEM) study. For the (6% Ag)-CuI thin film: (a, b) bright-field images at varying magnifications, (c) high-resolution TEM (HRTEM) image, and (d) selected area electron diffraction (SAED) pattern. TEM images of the (12% Ag)-CuI thin film: (e, f) bright-field images at varying magnifications, (g) HRTEM image, and (h) SAED pattern.

To further understand the SILAR method-derived morphology and impact of Ag addition, transmission electron microscopy (TEM) was performed with Ag 6% and Ag 12%. Figure 4a,b depicts TEM bright-field images of the Ag (6%)-doped CuI thin film sample, revealing colloidal nanoparticles of CuI with consistent sizing and effective deposition via the SILAR technique onto a glass substrate. Analysis of these TEM images suggests that the synthesized CuI nanoparticles range in size from 5 to 20 nm; however, they exhibit poor separation, forming colloidal aggregates indicative of their fine and porous nature, displaying cloud-like structures with interparticle porosity. Moreover, high-resolution TEM imaging reveals cross-sectional views of the (111) crystalline plane with a lattice spacing of 0.317 nm, marginally reduced due to Ag addition, signifying a cubic γ-CuI (111) configuration, as depicted in Figure 4c.^38^ The absence of discernible AgI or Ag in high-resolution TEM further indicates the absence of composite formations within this thin film, suggesting substantial incorporation of Ag within CuI. Furthermore, the SAED pattern corroborates the dominance of the (111) crystalline plane in the cubic γ-CuI structure (Figure 4d), affirming the high crystallinity of the samples and aligning with the XRD findings.

Increasing the Ag incorporation from 6 to 12% in CuI leads to significant changes in both the morphology and phase formation of the material. TEM analysis reveals two distinct colloidal morphological distributions—sheet-like structures and clusters—shown in Figure 4e,f. Further HRTEM analysis indicates that the crystalline lattice fringes are a mixture of both β-AgI and γ-CuI phases. The sheet-like structures exhibit a *d*-value of 0.56 nm, suggesting a more dominant presence of β-AgI, while the clusters have a *d*-value closer to 0.42 nm, indicative of more γ-CuI appearance. This suggests that Cu and Ag exhibit complete miscibility during alloy formation, as no distinct peaks corresponding to separate AgI or CuI phases are observed in Figure 2. The merging of these phases into a single alloy structure highlights the absence of any doping effects, as shown in Figure 4g. Additionally, the crystalline phase analysis indicates that the phase is predominantly β-AgI, as shown in Figure 4h. During the TEM analysis, the presence of cluster-type disordered hierarchical structures (Figure 3g) was observed in greater detail, revealing a colloidal porous network. Notably, a subtle morphological transformation was identified, which is characterized by an intensified distribution of the porous network. This transformation became more pronounced as the Ag concentration increased from 6 to 12%, resulting in the emergence of two distinct segments. Thus, the TEM analysis suggests that the morphological distribution and phase formation in 6 and 12% Ag–CuI could significantly influence their TE properties.

**Figure 5:**
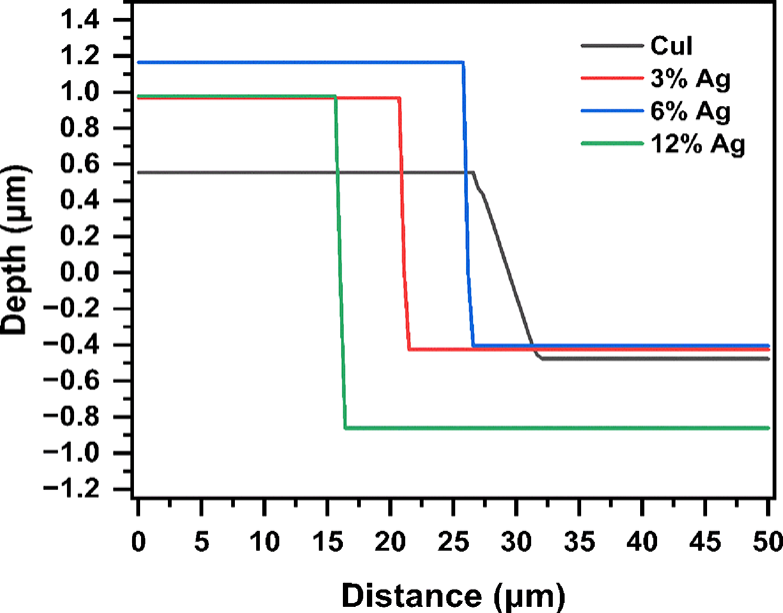
AFM profile depth analysis plot of various Ag–CuI thin film samples.

To accurately determine the thickness of the thin films, AFM depth profiling was performed. Figure 5 illustrates the AFM depth measurements of the films. The CuI thin film exhibited a thickness of approximately 1 μm. With the introduction of Ag doping, the thickness increased, reaching 1.4 μm. Additionally, the film containing 6% Ag showed a further increase in thickness up to 1.57 μm. As the addition of Ag increased to 12%, the average film thickness increased to 1.9 μm. These results indicate that the increase in Ag content leads to a corresponding increase in film thickness, likely due to the enhanced reaction between Ag and iodide.

To further verify the presence of Ag doping, XPS analysis was conducted to investigate the chemical composition of the thin film. The XPS analysis of the 6 and 12% Ag-doped CuI thin film samples revealed an intriguing chemical composition, indicating a transition from doping to alloy formation.

**Figure 6:**
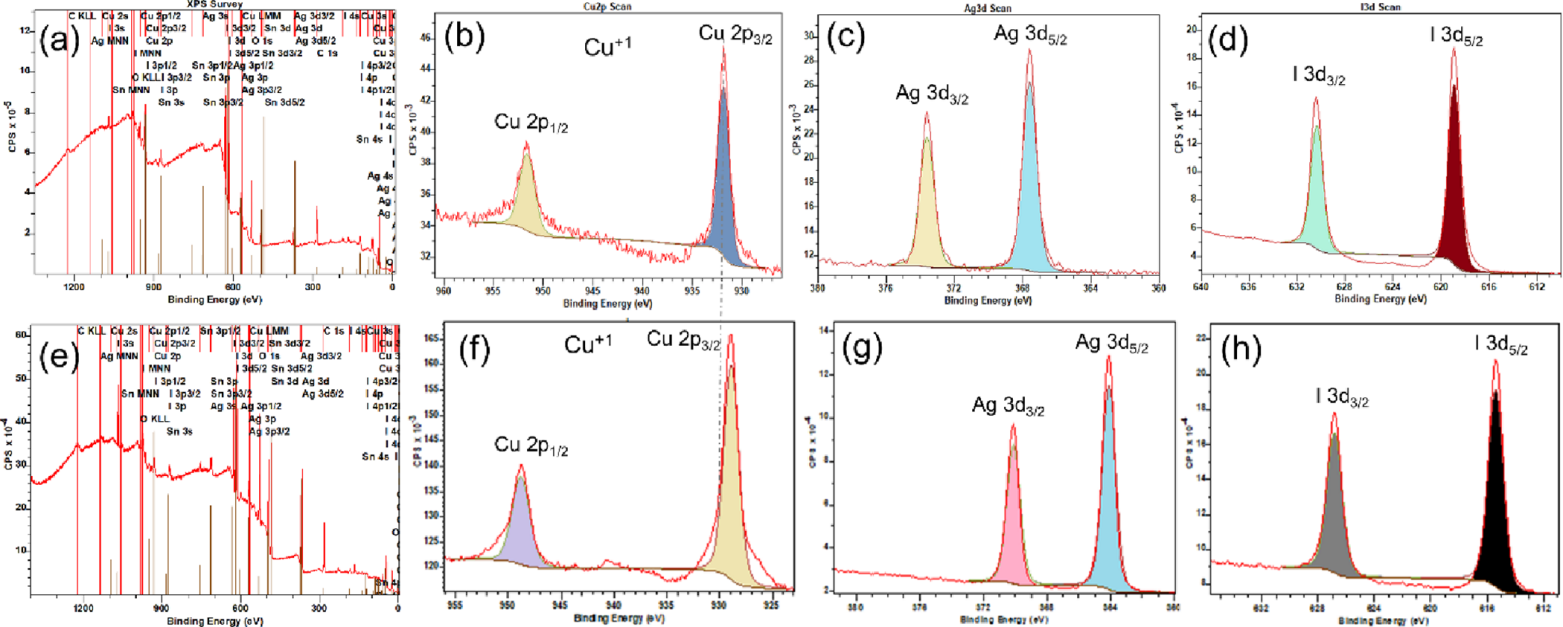
(a) X-ray photoelectron spectroscopy (XPS) survey spectrum for the CuI thin film sample doped with 6% Ag. Core-level spectra for (b) Cu 2p, (c) Ag 3d, and (d) I 3d for the same sample. (e) XPS survey spectrum for the CuI thin film sample incorporation with 12% Ag. Core-level spectra for (f) Cu 2p, (g) Ag 3d, and (h) I 3d for the same sample.

For the 6% Ag-doped CuI thin film sample, the XPS survey spectrum identified Cu, Ag, I, and O as the predominant elements within the sample, with trace amounts (<5%) of additional elements likely originating from the glass substrate (Figure 6a). The core-level spectrum of Cu 2p revealed binding energies of Cu 2p_3/2_ and 2p_1/2_ at 932.02 and 951.83 eV, respectively, corresponding to the Cu^1+^ oxidation state (Figure 6b). Notably, there were no discernible binding energy peaks near 935 and 955 eV, indicating the absence of Cu^2+^ and confirming the predominant presence of Cu^1+^ species.^39^ The Ag 3d_5/2_ and Ag 3d_3/2_ peaks were observed at 368.09 and 374.08 eV, respectively, consistent with the Ag^1+^ oxidation state in the thin film sample, thus confirming the doping within the CuI matrix (Figure 6c).^22^ Additionally, the binding energies of I 3d_5/2_ and 3d_3/2_ at 619.2 and 630.7 eV, respectively, indicated the presence of iodine in the +1-oxidation state (Figure 6d).

Interestingly, doubling the Ag addition level to 12% resulted in significant changes in the binding energies. The survey spectrum of the 12% Ag–CuI thin film sample confirmed the presence of Cu, Ag, and I, with no evidence of other materials or contamination, while the presence of Sn and O peaks indicated deposition on an FTO substrate (Figure 6e). The core-level spectra of Cu 2p_3/2_ and 2p_1/2_ for the 12% Ag-addition sample were determined to be at 928.80 and 948.82 eV, respectively (Figure 6f). Additionally, the Ag^1+^ oxidation state in this sample showed a notable red shift in the core-level spectrum compared to the 6% Ag-doped sample, approximately 1.5% (Figure 6g). Conversely, the I 3d_5/2_ and 3d_3/2_ binding energies at 615.34 and 628.86 eV, respectively, exhibited a significant blue shift compared to the 6% Ag-doped sample (Figure 6h). Similarly, the full width at half-maximum (FWHM) of the core-level spectrum peaks of 12% Ag incorporation thin samples has been reduced by an average of 3% compared to the 6% Ag-doped CuI thin film. This indicates a reduction in the crystallite size, which is consistent with the XRD results.

Doubling the Ag content in CuI results in a noticeable peak shift for both Cu and Ag–I, with the shift predominantly toward the higher end of the spectrum. This minor red shift is mainly attributed to the higher electronegativity of Ag (1.930) compared to Cu (1.90), which increases the electron density around CuI, leading to the observed red shift. Additionally, iodine plays a crucial role in determining the CuAgI alloy structure, as observed in both the XRD and TEM analyses. The formation of interstitial iodine defects and the creation of a metal-bridging dimer between a lattice iodine and interstitial iodine upon the capture of a hole by the interstitial iodine contribute significantly to this red shift.^40^ This combined effect of increased electron density and iodine-related defects results in a red shift of the material. Moreover, it is anticipated that Ag–Cu bimetallic composites can exhibit distinct plasmonic responses that are influenced by the grain or unit size of the material. The plasmonic behavior of such nanocomposites and its dependence on morphological and structural factors remain an active area of scientific investigation. Therefore, in accordance with the above discussion, various characterizations were employed to investigate the morphology and the incorporation of Ag as a dopant into the CuI system.

EDS analysis of Ag–CuI thin films provided a foundation for the further quantitative evaluation of their chemical composition. The EDS characterization of the CuI thin film on an FTO substrate, summarized in Table 2, identified peaks corresponding to Cu and I elements. Specifically, the elemental ratio of I to Cu was observed to be 2, aligning with previously reported findings.^23,41,42^ Remarkably, the atomic percentage of Cu cations was lower than that of I anions, indicating the presence of Cu vacancies. These vacancies play a crucial role in enabling the p-type semiconductor properties of CuI.^17^ While EDS cannot directly detect vacancies, the substoichiometric Cu content inferred from the elemental ratio supports the presence of Cu vacancies, which are known to enable the p-type semiconductor properties of CuI.

**Table 2:** EDS and AAS Analyses of Ag–CuI Thin Films with Varying Ag Concentrations

**EDS analysis**				**AAS analysis**	
**thin film**	**Ag** (wt %)	**Cu** (wt %)	I (wt %)	**Ag** (at. %)	**Cu** (at. %)
CuI		28.7	57.3		
Ag (3%)-CuI	2.7	24.8	52.4	0.07	0.93
Ag (6%)-CuI	7	19.5	47.8	0.2	0.8
Ag (12%)-CuI	29	1.9	36.3	0.9	0.1

The EDS analysis reveals the presence of a Ag peak when the Ag doping percentage is 3%, with the observed elemental weight ratio at approximately 2.7%. This weight ratio increases to 7% when the Ag doping level is increased to 6%. Interestingly, the iodine weight ratio remains around 50% even with increasing the level of Ag doping. However, when the Ag content reaches 12%, the weight ratio of I and Cu decreases, while the Ag weight ratio increases.

For further quantitative analysis, an AAS has been conducted to trace the Ag, Cu, and I elements in the samples, as shown in Table 2. It can be noticed that the element weight matches with the trend in the EDS and agrees with the XRD and SEM results. The Ag content in the films increases with the increase of Ag addition percentage from 0.07 at 3% Ag to 0.9 at 12% Ag.

**Figure 7:**
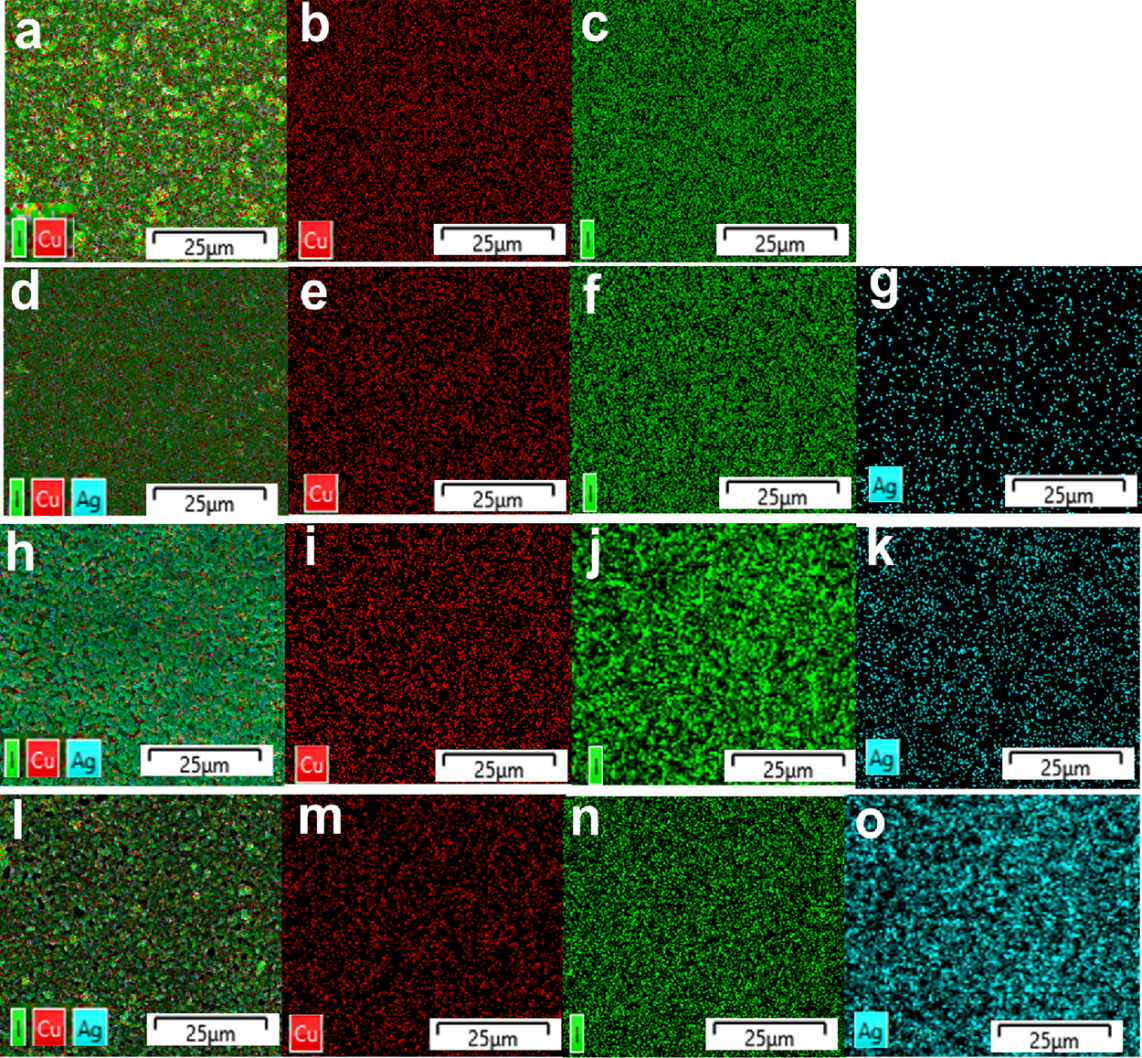
X-ray energy-dispersive spectroscopy (EDS) elemental color mapping for the CuI thin film: (a–c), Ag_0.07_Cu_0.93_I thin film (d–g), Ag_0.2_Cu_0.8_I thin film (h–k), and Ag_0.9_Cu_0.1_I thin film (l–o).

Figure 7 presents the EDS elemental color mapping of the analyzed thin films. The elemental distribution appears uniformly dispersed across all samples, with iodine consistently exhibiting the highest concentration. In the CuI thin film (Figure 7a–c), a pronounced enrichment of iodine is observed with an intensity nearly double that of Cu. For the Ag_0.07_Cu_0.93_I thin film (Figure 7d–g), the uniform distribution of Ag confirms the successful incorporation of Ag through doping. Similarly, in the Ag_0.2_Cu_0.8_I thin film (Figure 7h–k), the increased Ag concentration corresponds to the higher Ag content in the film. Finally, in the Ag_0.9_Cu_0.1_I thin film (Figure 7l–p), the Ag concentration exceeds that of Cu, confirming the dominance of Ag and supporting the phase transition to AgI.

### 3.2 Optical Characterizations of Ag–Cu–I Thin Films

**Figure 8:**
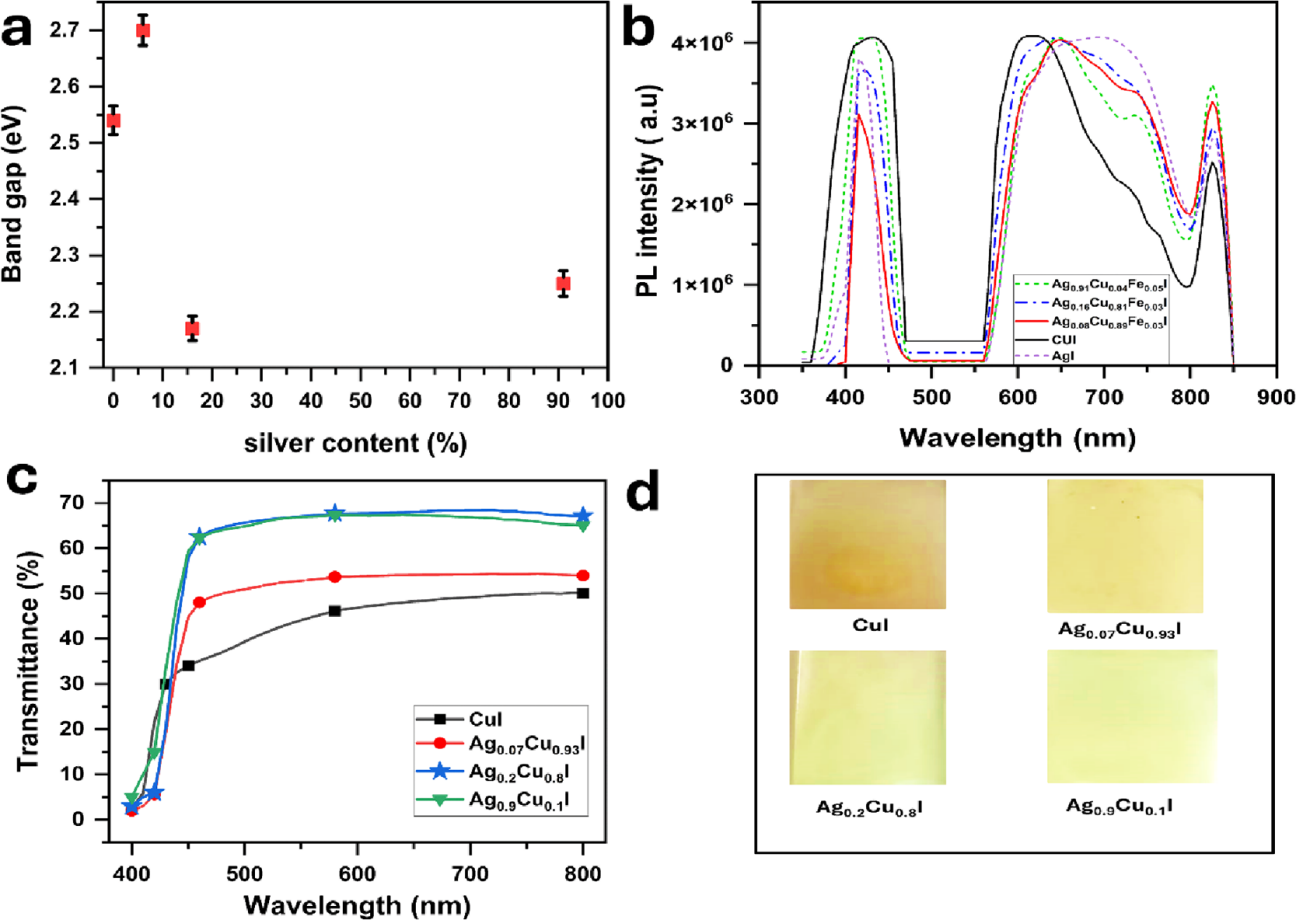
(a) Optical band gap of the Ag–CuI thin films containing different Ag levels. Error bars were calculated using OriginPro 2024 software based on the standard deviation of five independent measurements per sample. (b) Corresponding photoluminescence spectra. (c) Transmission spectra of the Ag–CuI thin films. (d) Digital photograph of Ag–CuI thin films.

To further understand the optical and electrical properties, a band gap measurement was conducted. For the CuI thin film, the optical band gap is approximately 2.54 eV (Figure 8a). The band gap starts to increase with addition of Ag and reaches 2.7 at 0.07 Ag content. This figure reduces to 2.17 eV for 0.2 Ag content. Increasing the Ag content to 0.9, the band gap increases to 2.25 eV. This narrow band gap of Ag incorporation compared to CuI matches with a previous report.^20^ This narrower band gap could be attributed to quantum confinement effects due to the shape of the Ag–CuI thin film that look like rods and tubes.^43,44^ This relatively low band gap could be a reason for the high result of *S*.

To explore the optical properties of Ag–CuI thin films, photoluminescence (PL) measurements were conducted at room temperature (Figure 8b). The PL spectra revealed three primary bands located in the blue, red, and infrared regions. The blue band displayed a sharp peak around 415 nm, while the red band was broader, centered at approximately 730 nm. The narrowest band in the infrared region was centered near 825 nm. These bands align with previously reported findings.^45^ The blue band and its shoulder in CuI are attributed to radiative excitonic recombination and transitions from the conduction band to Cu-vacancy defect states, respectively. The red band’s PL arises from transitions from iodine-vacancy defect states to the valence band. Notably, the relative spectral features of the blue band are influenced by the Ag content. As Ag content increases, the PL intensity of the blue band rises, accompanied by a red shift. Conversely, the red band exhibits a blue shift with increasing Ag content.

The optical transmittance of Ag–CuI thin films deposited on FTO with varying Ag contents, measured in the 400–800 nm wavelength range, is presented in Figure 8c. The average transmittance of the film ranges from 50 to 68% in the visible spectrum. Notably, the transmittance increases with increasing Ag content, peaking at 68% for a Ag concentration of 0.2, before slightly decreasing to 67% for a Ag concentration of 0.9. This trend aligns with a previous report.^20^ Additionally, the color of the CuI thin films is yellow, but as the Ag content increases, the color shifts from yellow to yellow-green and ultimately to green at higher Ag concentrations, as shown in Figure 8d.

### 3.3 Thermoelectric Performance Analysis

**Figure 9:**
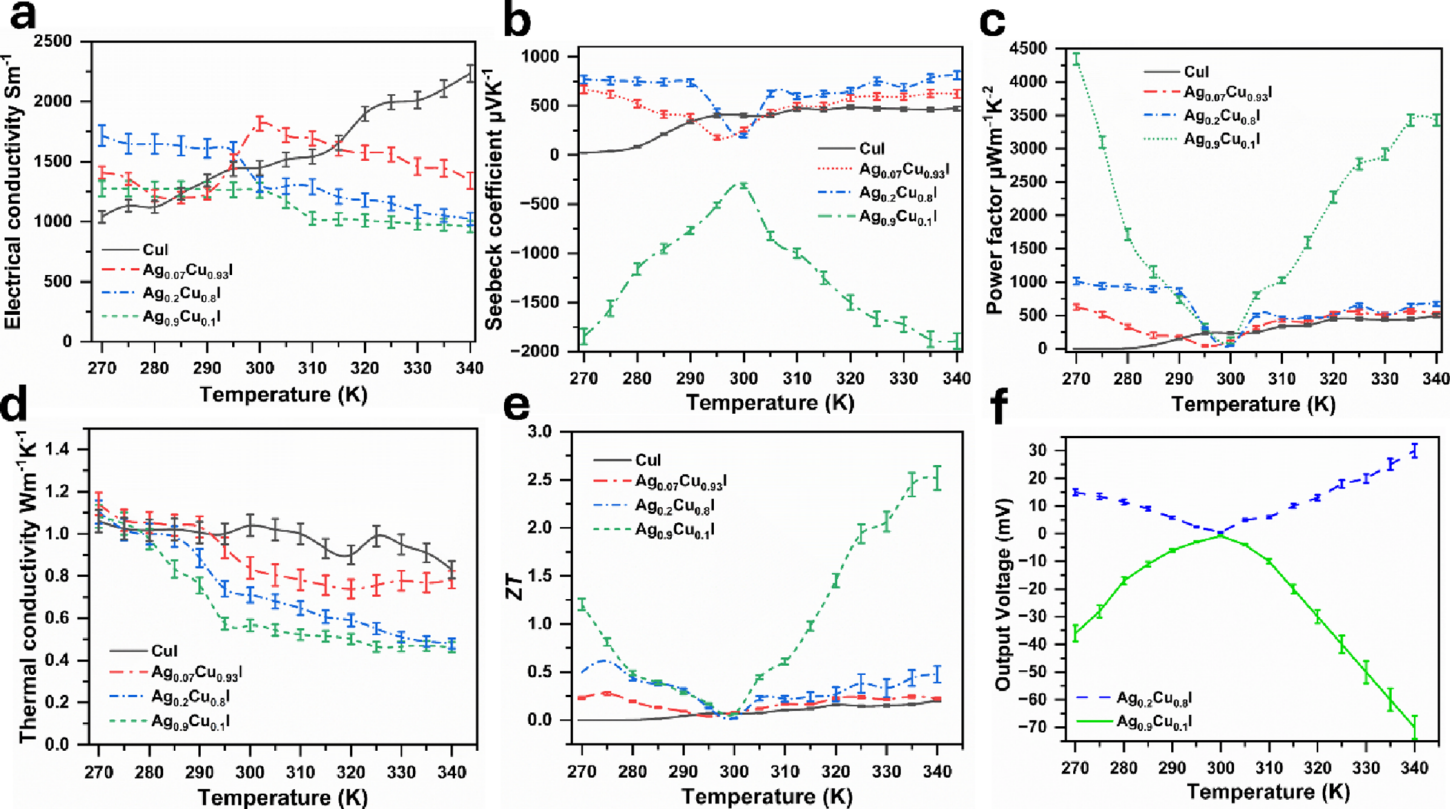
Thermoelectric properties of Ag–CuI thin film devices deposited on FTO glass. (a) Electrical conductivity. (b) Seebeck coefficient. (c) Thermoelectric power factor. (d) Thermal conductivity. (e) *ZT*. (f) Thermoelectric output voltage of the best performance samples. Error bars were calculated by using OriginPro 2024 software based on the standard deviation (SD) of five independent measurements per temperature point.

To understand the TE performance of Ag–CuI thin films, σ, *S*, and *k* were measured and *PF* and *ZT* were calculated. The σ of the Ag–CuI thin film samples is illustrated in Figure 9a. The σ of CuI increased with temperature from 1040 Sm^–1^ at 270 K to 2233 Sm^–1^ at 340 K. For Ag_0.07_Cu_0.93_I, σ oscillated below room temperature, reduced from 1407 Sm^-1^ at 270 K to 1234 Sm^-1^ at 290 K, and then increased to 1816 Sm^-1^ at 300 K and reduced with temperature above room temperature to 1340 Sm^–1^ at 340 K. Moreover, the σ reduced with the increase of Ag incorporation. When the Ag addition increased at sample Ag_0.2_Cu_0.8_I, σ reduced with temperature from 1715 Sm^–1^ at 270 K to 1023 Sm^–1^ at 340 K. With further increase in Ag incorporation in sample Ag_0.9_Cu_0.1_I, σ reduced with temperature from 1275 Sm^–1^ at 270 to 958 at 340 K. The σ of the films increased with temperature for CuI but decreased with temperature when Ag was introduced. This behavior is attributed to ionized impurity and phonon scattering. The presence of Ag acts as a scattering center, and with increasing temperature, lattice vibrations intensify, further reducing carrier mobility.

The *S* of the Ag–CuI thin films is illustrated in Figure 9b. The *S* of undoped CuI measured around 24 μV K^-1^ at 270 K, and it increased with temperature up to 473 μV K^-1^ at 340 K. Moreover, the samples with Ag addition to CuI followed the same pattern; they increased above room temperature and reduced below room temperature. For Ag_0.07_Cu_0.93_I, *S* increased to 616 at 270 K and then it started to reduce until room temperature and then increased with temperature to 621 μV K^–1^ at 340 K. Further increasing Ag addition, the *S* coefficient of Ag_0.2_Cu_0.8_I reached around 810 μV K^–1^ at 340 K. Additionally, when the Ag incorporation reached 0.9, the material behavior changed from p-type to n-type and the *S* coefficient reached around −1846 μV K^–1^ at 270 K, −312 μV K^–1^ at 300 K, and then increased up to −1891 μV K^–1^ at 340 K.^46^ This change of behavior from p-type to n-type is due to a change in materials from γ-CuI to β-AgI, which has been confirmed by the characterization techniques. It is noticed that *S* of the thin films increased with increasing temperature. Usually, TE materials exhibit an increase in *S* with temperature, as observed in pure CuI. However, an interesting finding in this research is that materials with Ag incorporation show a high *S* even at low temperature differences. This suggests that the presence of Ag may introduce impurity states that enhance carrier asymmetry, resulting in higher *S* at lower temperatures.

The PF of thin films is demonstrated in Figure 9c. The PF of CuI reached 500 μW m^–1^ K^-2^ at 340 K. This figure slightly increased to 517 μW m^–1^ K^–2^ at 340 K when it doped with Ag. Further increasing Ag addition for the sample Ag_0.2_Cu_0.8_I, the PF increased and reached 672 μW m^–1^ K^–2^ at 340 K. Additionally, when the Ag content reached 0.9, it resulted in a high PF by reaching up to 3428 μW m^–1^ K^–2^ at 340 K. This high PF of Ag_0.9_Cu_0.1_I thin film is a result of high σ and ultrahigh *S*.

Figure 9d shows parts *k* of the Ag–CuI thin films. *k* of the samples reduces with increasing the temperature. The *k* of CuI is usually around 0.5 W m^–1^ K^–1^;^12^ however, the CuI thin film sample has a higher value around 1.06 W m^–1^ K^–1^ at 270 K. This is due to the conductive substrate (FTO), which has a *k* of 1.17 W m^–1^ K^–1^. The *k* of CuI reduced with temperature to 0.83 W m^–1^ K^–1^ at 340 K. Moreover, *k* reduced with increasing Ag content.^22^ When the Ag content reached 0.07, *k* reduced to 0.78 W m^–1^ K^–1^ at 340 K. Moreover, when the Ag content reached 0.2, *k* reduced from 1.1 W m^–1^ K^–1^ at 270 K to 0.48 W m^–1^ K^–1^ at 340 K. One of the reasons for this low value is the structure shape of the particles, which looked like rods. Nanorods increase the surface to volume area and increase the photon scattering. Moreover, doping plays a big role as defects and the defects in turn reduce *k.*(47) Finally, when the Ag content reached 0.9, *k* showed an ultralow value of 0.46 W m^–1^ K^–1^ at 340 K; this is due to the nanostructure of the Ag_0.9_Cu_0.1_I, which looked like nanotubes, as shown in the SEM images. This nanotube structure increases the phonon scattering, which in turn reduces *k.*(48)

Figure 9e illustrates the *ZT* value for the Ag–CuI thin film samples. The *ZT* of CuI was around 0.2 at 340 K. This figure slightly increased with increasing Ag content to 0.07 to 0.22. Moreover, the *ZT* value further increased with an increase of Ag content and reached 0.47 at 340 K when the Ag content was 0.2. Finally, a high *ZT* was achieved when the Ag content increased to 0.9 and reached around 2.5 at 340 K. This high *ZT* value is due to the high PF and low *k*.

Figure 9f shows the TE output voltage of the best samples that have been chosen according to their TE performance. It is noticed that Ag_0.2_Cu_0.8_I is p-type as it has a positive voltage and Ag_0.9_Cu_0.1_I is n-type as it has a negative voltage. The TE output voltage of Ag_0.2_Cu_0.8_I reached around 20 mV at 270 K and 30 mV at 340 K. Moreover, the Ag_0.9_Cu_0.1_I thin film reached −36 mV at 270 K and −70 mV at 340 K. This interesting finding of producing high voltage at small temperature differences at low temperature leads to a high *ZT* at 270 K, indicating that this material can perform efficiently not only in hot environments but also in cool environments. This expands its potential applications, making it suitable for energy harvesting in diverse temperature conditions.

Table 3 presents a comparative analysis of the findings from this study against previously reported literature, highlighting a marked improvement in performance. The results unequivocally demonstrate that the material developed in this work not only surpasses the performance of CuI-based systems in prior studies but also exhibits competitiveness with conventional TE materials.

**Table 3:** Thermoelectric Performance Comparison between Ag_0.2_Cu_0.8_I and Ag_0.9_Cu_0._1I, which Has Been Developed in This Work and a Previous Reported Work

no.	material	electrical conductivity (S m^–^^1^)	Seebeck coefficient (μV K^-1^)	power factor (μW m^-1^ K^-2^)	synthesis method	reference
**1**	CuI-doped Tb	700	550	211	precipitation method	(41)
**2**	CuI	11000	206	470	resistive thermal evaporation	(17)
**3**	CuI	575	108	6.7	SILAR	(49)
**4**	Ag-doped CuI	360	400	26	chemical route	(22)
**5**	Bi_0.5_Sb_1.5_Te_3_	650	242	38	spark plasma sintering	(50)
**6**	Te-embedded Bi_2_Te_3_	825	–234	45.2	molecular beam epitaxial	(51)
**7**	Ag_0.2_Cu_0.8_I	1023	810	672	SILAR	this work
**8**	Ag_0.9_Cu_0.1_I	958	–1891	3428	SILAR	this work

**Figure 10:**
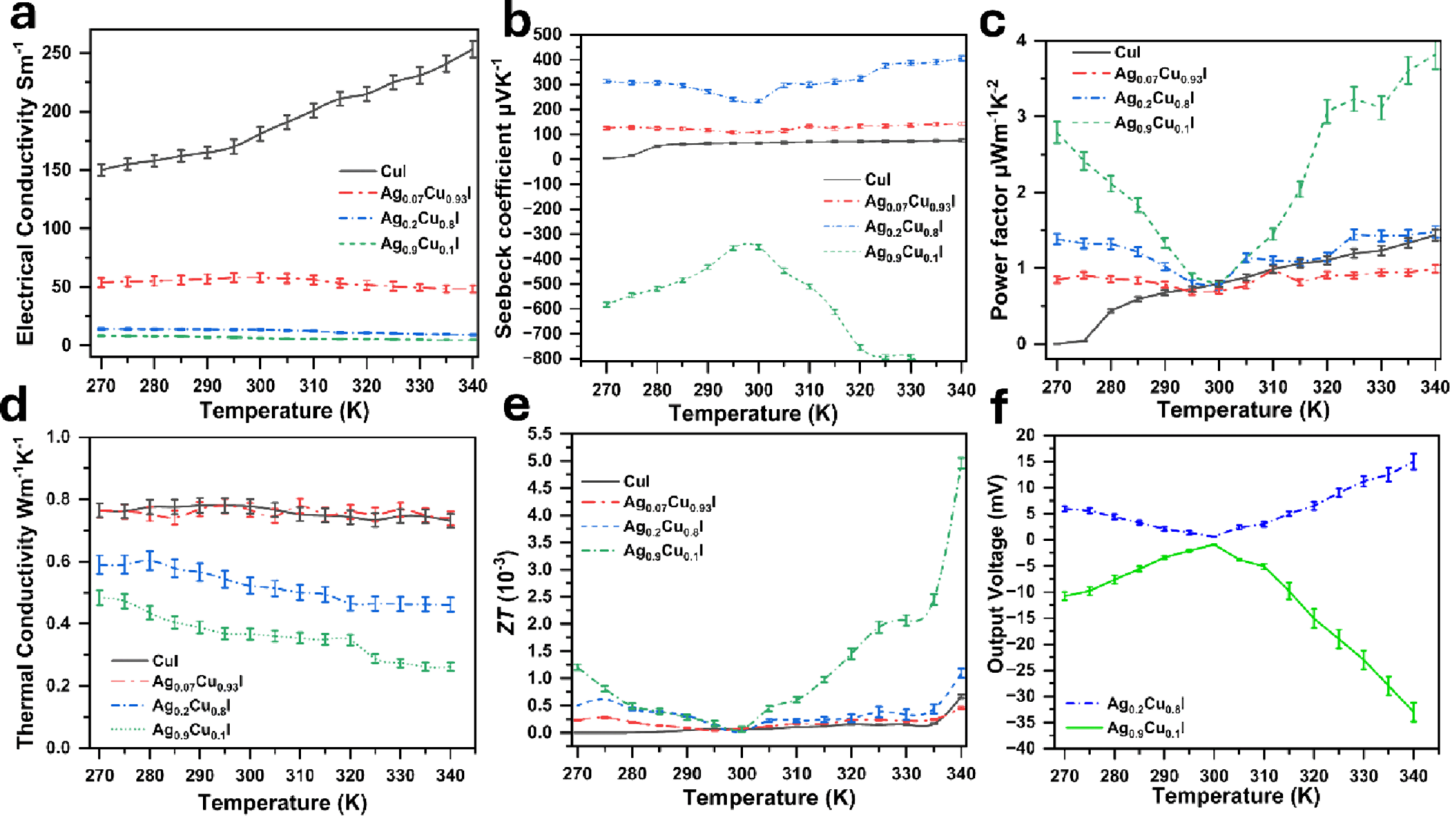
Thermoelectric properties of Ag–CuI thin film samples deposited on glass. (a) Electrical conductivity. (b) Seebeck coefficient. (c) Thermoelectric power factor. (d) Thermal conductivity. (e) *ZT*. (F) Thermoelectric output voltage of the best samples (Ag_0.2_Cu_0.8_I, Ag_0.9_Cu_0.1_I). Error bars were calculated using OriginPro 2024 software based on the standard deviation of five independent measurements per temperature point.

To further investigate the effect of the FTO substrate on the TE properties, the thin film was deposited on normal glass, and its TE properties, were measured and calculated. The results are presented in Figure 10, highlighting the influence of the substrate on the material’s performance. It can be seen in Figure 10a that σ was reduced 10 times than the samples deposited on FTO. Moreover, the *S* of the samples was also reduced as shown in Figure 10b. PF was also reduced due to reduction in σ, *S* as shown in Figure 10c. However, *k* was enhanced and showed higher reduction compared to the samples deposited on the FTO, as shown in Figure 10d. For example, the *k* of Ag_0.9_Cu_0.1_I reached an ultralow *k* of 0.26 W m^–1^ K^–1^. Furthermore, *ZT* was reduced 500 times compared to the samples deposited on FTO, indicating a large contribution of FTO on the TE performance of the thin films (Figure 10e). Figure 10f shows the output voltage of thin films reduced by half compared with the samples deposited on FTO.

**Figure 11:**
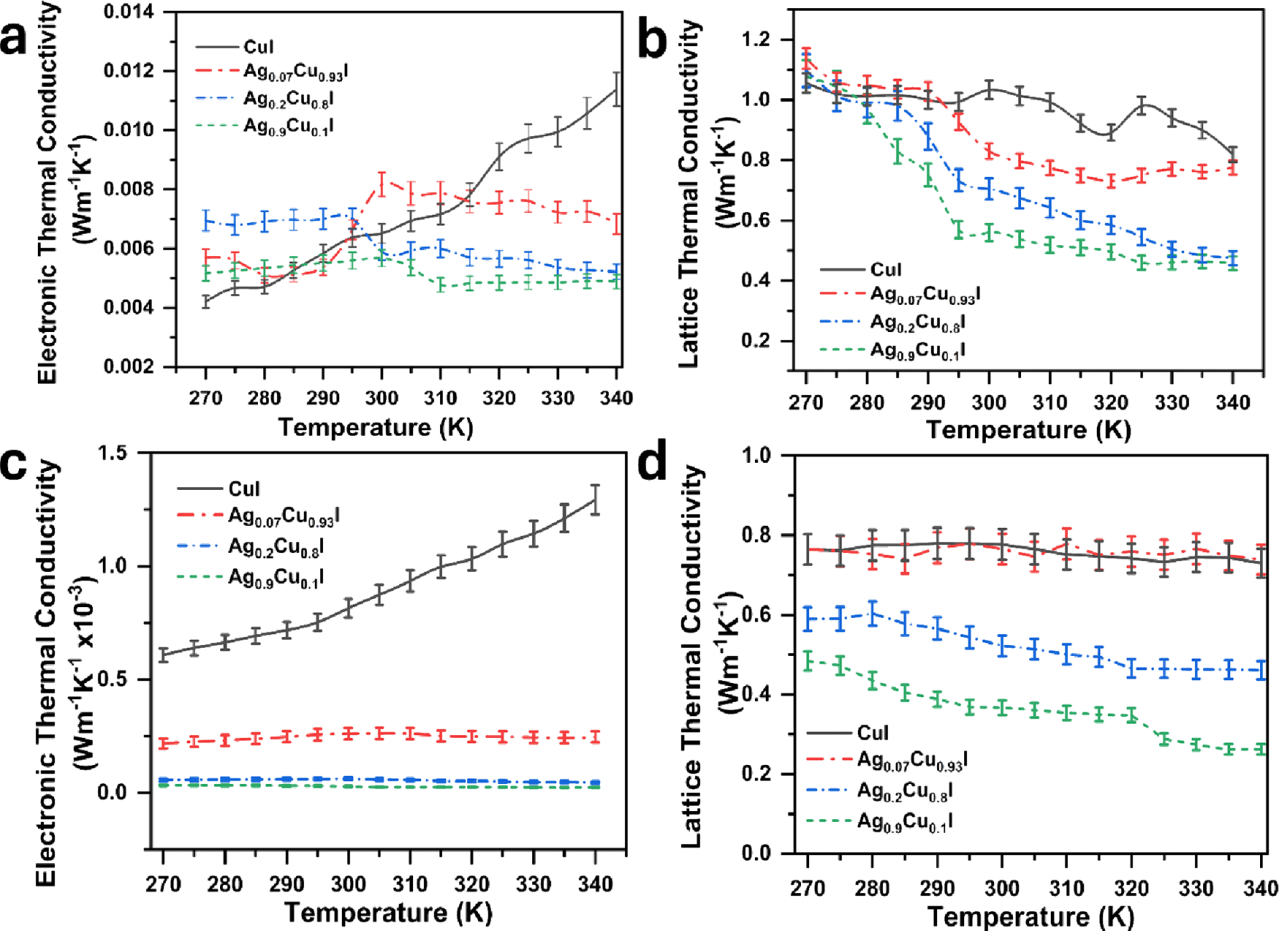
Temperature dependence of electronic and lattice thermal conductivity of Ag–CuI. (a) Electronic thermal conductivity of samples deposited on the FTO. (b) Lattice thermal conductivity of samples deposited on the FTO. (c) Electronic thermal conductivity of samples deposited on glass. (d) Lattice thermal conductivity of samples deposited on the glass. Error bars were calculated using OriginPro 2024 software based on the standard deviation of five independent measurements per temperature point.

For further investigation of the substrate’s effect on *k*, both *k*_e_ and *k*_l_ were calculated according to eqs 1 and 2, with results presented in Figure 11. The data show that films deposited on FTO exhibit higher *k*_e_, which may contribute to an overall increase in the total *k* (Figure 11a,b). Notably, the *k* value of CuI films is most significantly influenced by *k*_e_. In contrast, the samples deposited on glass show a reduced impact on the thermal conductivity (Figure 11c,d).

### 3.4 Performance Analysis of the Thermoelectric Glazing Prototype

**Figure 12:**
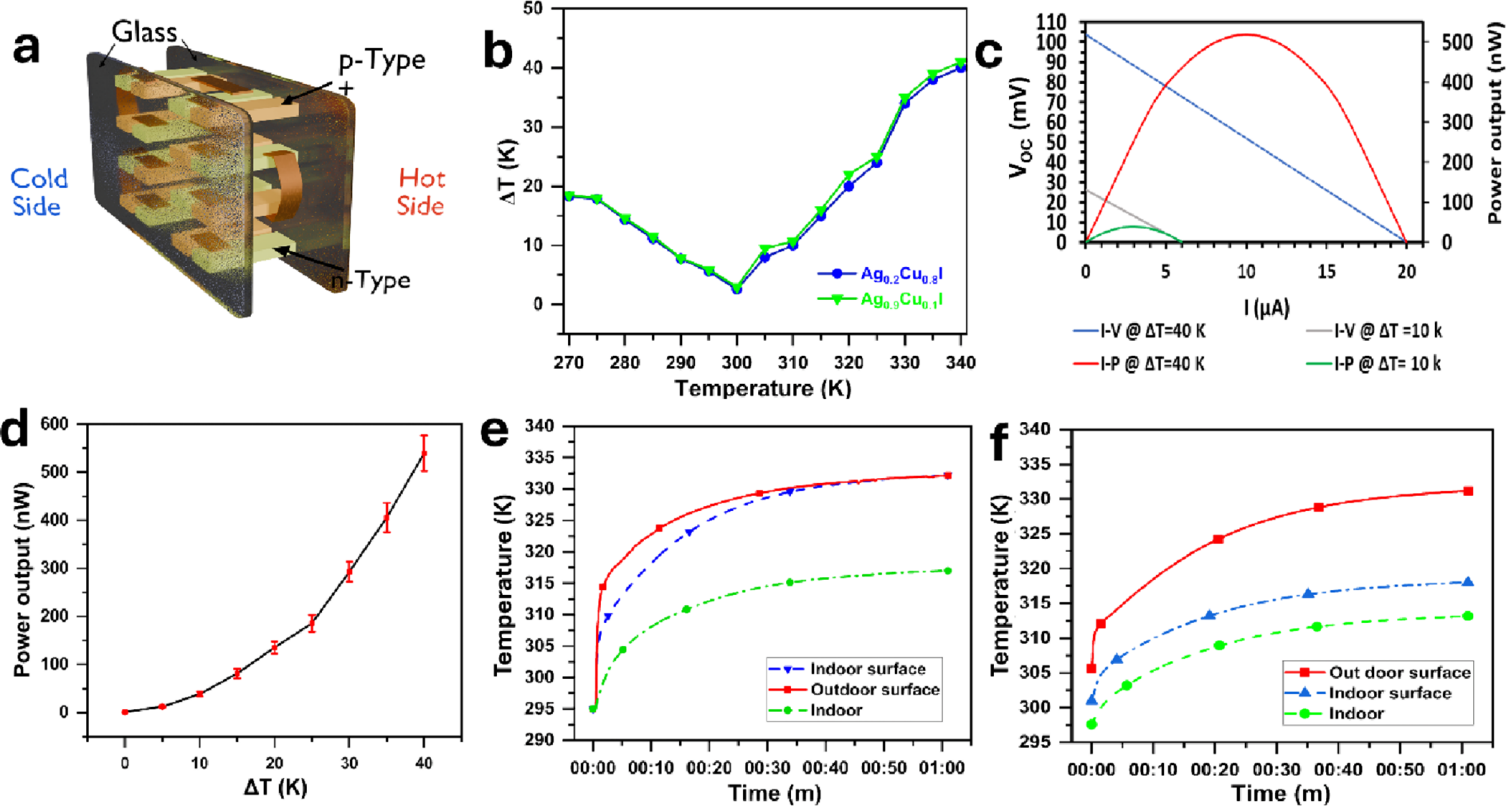
Thermoelectric glazing performance analysis. (a) Illustration of the thermoelectric glazing design, which consists of eight nanogenerators. Each nanogenerator has p-type and n-type deposited on FTO glass. (b) Measuring temperature differences along thin films with increase of temperature. (c) Calculated current–voltage (*I*–*V*) and current–power (*I*–*P*) at 10 and 40 K temperature differences. (d) Measured output power with temperature differences. The temperature profile of (e) thermoelectric glazing. (f) double glazing with glass. Error bars were calculated using OriginPro 2024 software based on the standard deviation of five independent measurements per temperature point.

The TEGZ prototype features a double-glazing configuration with an area of 5 × 5 cm^2^ per glass panel, within which TEGs are embedded, as shown in Figure 12a. The TEGs consist of thin films deposited on FTO substrates, each measuring 2 cm in length and 1 cm in width. These TEGs are composed of both p- and n-type TE materials, with the highest TE performance achieved by p-type (Ag_0.2_Cu_0.8_I) and n-type (Ag_0.9_Cu_0.1_I) materials. Cu tape is employed as a conductive electrode to interconnect the p-type and n-type materials. This configuration enables the establishment of a high-temperature gradient between the cold and hot sides of the device. The temperature difference arises from in-plane heat transfer along the length of the samples (2 cm), rather than from the cross-plane heat transfer characteristic of the thin film samples (1 μm). This configuration minimizes heat loss and enhances the efficiency of heat-to-electricity conversion, as the TE output voltage increases with both the temperature and the temperature gradient.

Figure 12b illustrates the temperature differences observed for the Ag_0.2_Cu_0.8_I and Ag_0.9_Cu_0.1_I samples as the temperature increases. At 340 K, the temperature difference across the length of the thin films was 40 K for Ag_0.2_Cu_0.8_I and 41 K for Ag_0.9_Cu_0.1_I, owing to the lower *k* of Ag_0.9_Cu_0.1_I compared to that of Ag_0.2_Cu_0.8_I. The TEGZ device consists of eight TEGs arranged in four rows, with each row containing two TEGs connected electrically in series and thermally in parallel. These rows are electrically connected in parallel. The performance of one nanogenerator was evaluated over a 40 K temperature difference. Cu tape was used for interconnection between the p- and n-type materials, as depicted in Figure 12a.

The output power of the nanogenerator was calculated as shown in Figure 12c, following the measurement procedure outlined in the device design (Figure 1d). The results show that at a temperature difference of 10 K, the open-circuit voltage (*V*_OC_) was approximately 26 mV, the short-circuit current was 6 μA, and the output power was around 39 nW. As the temperature difference increased to 40 K, the output power rose to 520 nW, with the *V*_OC_ increasing to approximately 104 mV and the short-circuit current rising to 20 μA. The measured output powers at 10 and 40 K temperature differences were approximately 38 and 540 nW, respectively (Figure 12d), closely matching the calculated output power.

Table 4 illustrates the reported TEG output. It can be seen clearly that TEG in this work overcomes TEG made from state-of-the-art materials such as CuI. Moreover, the TEG in this work has high-power output than traditional high-efficiency materials. For example, one pair of Ag_0.12_Cu_0.88_I and Ag_0.06_Cu_0.94_I is equal to 520 nW while (10 pairs) of Bi_2_Te_3_ and Sb_2_Te_3_ has only 10 nW. This high value comes from high output voltage 104 mV compared to 17.7 mV reported in ref (52).

**Table 4:** Comparison of the Thermoelectric Nanogenerator Performance

	material				
no.	n-type	p-type	output voltage (mV)	output power (nW)	reference
1	Bi_1.75_Te_3.25_	Bi_0.4_Sb_1.6_Te_3_	10	160 (72 pairs)	(14)
2	Bi_2_Te_3_	Sb_2_Te_3_	17.7	16 (1 pair)	(52)
3		CuI	2.5	8.2 (1 pair)	(12)
4	GZO	CuI	3	0.046 (1 pair)	(17)
5	Bi_2_Te_3_	Sb_2_Te_3_	430 (100 pairs)	320 (100 pairs)	(53)
6	Bi_0.4_Sb_1.6_Te_3_	Bi_2_Se_0.3_Te_2.7_	55.15 (10 pairs)	4440 (10 pairs)	(54)
7	Bi_2_Te_3_	Sb_*x*_Te_*y*_	308.8 (71 pairs)	166 (71 pairs)	(55)
8	Ag_0.9_Cu_0.1_I	Ag_0.2_Cu_0.8_I	104	520 (1 pair)	this work

The TEGZ serves a dual purpose, functioning both as a generator and to reduce heat losses. To evaluate its effectiveness in minimizing thermal losses, a temperature profile study was conducted based on the setup described in refs (24,56). The TEGZ, measuring 5 cm × 5 cm^2^, was positioned at the center of an insulated box. Temperature measurements were recorded using three K-type thermocouples. Two thermocouples were strategically placed at the core of the TEGZ on opposite sides to measure internal and external surface temperatures, while the third was positioned inside the box, behind the TEGZ, to measure the indoor temperature. The heat source for the study was a light setup calibrated to 1 SUN and 1.5 AM.

Figure 12e exhibits the profile temperature of double glazing with time to compare it with the performance of reducing heat losses of TEGZ (Figure 12f.) In double glazing, the outside surface and inside surface have the same temperature after 30 min. In the case of TEGZ, there is a 14 K temperature difference between the outside surface and the inside surface. The indoor temperature of TEGZ is 312 K, which is less than that of double glazing by 4 K. This little temperature difference despite the huge difference between the outside surface and the inside surface is because of the TEGZ design as strips and it does not cover all the glazing. Thus, the heat passes between the TEGZ strips.

## 4 Conclusions

This study demonstrates the synthesis of high-performance p-type and n-type CuI-based TE materials using a cost-effective, greener, and scalable method called SILAR. By optimizing Ag doping content through a single synthesis route using the SILAR method, we successfully developed both p-type (Ag_0.2_Cu_0.8_I) and n-type (Ag_0.9_Cu_0.1_I) materials. The p-type material achieved a figure of merit (ZT) of 0.47 at 340 K, driven by a high-PF and low thermal conductivity (*k*). In contrast, the n-type material exhibited an exceptional ZT of 2.5 at 340 K, attributed to its extremely high Seebeck coefficient (*S*) and ultralow *k*. These superior properties are linked to the nanostructured morphology, point defects, narrow band gap, and contributions from the conductive glass.

These high-efficiency CuI-based materials present a compelling alternative to conventional TE materials with broad potential applications. A fabricated thermoelectric glazing nanogenerator (TEGZ) produced an output power of 520 nW at 340 K under a temperature gradient of approximately 40 K. Additionally, a 5 × 5 cm^2^ TEGZ prototype exhibited a temperature difference of 14 K between its external and internal surfaces, demonstrating dual functionality in TE energy conversion and effective heat loss mitigation. These results highlight the potential of TEGZ technology to advance energy-positive building designs, enabling simultaneous energy generation and conservation.
